# Roles of the RON3 C-terminal fragment in erythrocyte invasion and blood-stage parasite proliferation in *Plasmodium falciparum*

**DOI:** 10.3389/fcimb.2023.1197126

**Published:** 2023-06-29

**Authors:** Daisuke Ito, Yoko Kondo, Eizo Takashima, Hideyuki Iriko, Amporn Thongkukiatkul, Motomi Torii, Hitoshi Otsuki

**Affiliations:** ^1^ Division of Medical Zoology, Department of Microbiology and Immunology, Faculty of Medicine, Tottori University, Yonago, Japan; ^2^ Division of Malaria Research, Proteo-Science Center, Ehime University, Matsuyama, Japan; ^3^ Division of Global Infectious Diseases, Department of Public Health, Graduate School of Health Sciences, Kobe University, Kobe, Japan; ^4^ Department of Biology, Faculty of Science, Burapha University, Chonburi, Thailand; ^5^ Division of Molecular Parasitology, Proteo-Science Center, Ehime University, Toon, Japan

**Keywords:** malaria, *Plasmodium falciparum*, rhoptry, RON3, invasion, PVM, PTEX, nutrient uptake

## Abstract

*Plasmodium* species cause malaria, and in the instance of *Plasmodium falciparum* is responsible for a societal burden of over 600,000 deaths annually. The symptoms and pathology of malaria are due to intraerythocytic parasites. Erythrocyte invasion is mediated by the parasite merozoite stage, and is accompanied by the formation of a parasitophorous vacuolar membrane (PVM), within which the parasite develops. The merozoite apical rhoptry organelle contains various proteins that contribute to erythrocyte attachment and invasion. RON3, a rhoptry bulb membrane protein, undergoes protein processing and is discharged into the PVM during invasion. RON3-deficient parasites fail to develop beyond the intraerythrocytic ring stage, and protein export into erythrocytes by the *Plasmodium* translocon of exported proteins (PTEX) apparatus is abrogated, as well as glucose uptake into parasites. It is known that truncated N- and C-terminal RON3 fragments are present in rhoptries, but it is unclear which RON3 fragments contribute to protein export by PTEX and glucose uptake through the PVM. To investigate and distinguish the roles of the RON3 C-terminal fragment at distinct developmental stages, we used a C-terminus tag for conditional and post-translational control. We demonstrated that RON3 is essential for blood-stage parasite survival, and knockdown of RON3 C-terminal fragment expression from the early schizont stage induces a defect in erythrocyte invasion and the subsequent development of ring stage parasites. Protein processing of full-length RON3 was partially inhibited in the schizont stage, and the RON3 C-terminal fragment was abolished in subsequent ring-stage parasites compared to the RON3 N-terminal fragment. Protein export and glucose uptake were abrogated specifically in the late ring stage. Plasmodial surface anion channel (PSAC) activity was partially retained, facilitating small molecule traffic across the erythrocyte membrane. The knockdown of the RON3 C-terminal fragment after erythrocyte invasion did not alter parasite growth. These data suggest that the RON3 C-terminal fragment participates in erythrocyte invasion and serves an essential role in the progression of ring-stage parasite growth by the establishment of the nutrient-permeable channel in the PVM, accompanying the transport of ring-stage parasite protein from the plasma membrane to the PVM.

## 1 Introduction

Malaria is a life-threatening infectious disease in tropical and subtropical regions and is caused by parasites of the protozoan genus *Plasmodium*. The global tally of malaria cases reached 247 million, with an estimated 619,000 malaria deaths in 2021 (WHO, 2022). The clinical symptoms result from repeated amplification cycles of erythrocyte invasion by parasite merozoites, intraerythrocytic development, schizogony to form daughter merozoites, and rupture of the infected erythrocyte (Cowman et al., 2016). During erythrocyte recognition and invasion by merozoites, apical organelles unique to apicomplexan parasites, namely micronemes, rhoptries, and dense granules, are discharged and contain various proteins that play important roles (Cowman et al., 2017).

To initiate invasion, erythrocyte binding-like (EBL) and reticulocyte binding-like (RBL) proteins, localized in either micronemes or rhoptries, bind to erythrocyte surface receptors (Tham et al., 2012). Then the microneme protein AMA1, along with a rhoptry neck protein complex consisting of proteins RON2, RON4, and RON5, forms a tight junction between the erythrocyte and merozoite (Cao et al., 2009; Richard et al., 2010; Srinivasan et al., 2011; Sherling et al., 2019). The adhered parasite propels itself into the erythrocyte using an apicomplexan-unique process termed gliding motility, which is powered by a sub-membranous molecular motor apparatus called the glideosome (Cowman et al., 2017). As a consequence of erythrocyte invasion, the parasite envelopes and seals itself within a portion of the host membrane; and the resulting parasitophorous vacuole (PV) is created in part by the function of parasite molecules stored in apical organelles. The parasitophorous vacuolar membrane (PVM) is then modified by parasite molecules stored in the rhoptries and dense granules (Goldberg and Zimmerberg, 2020). For example, the rhoptry bulb low molecular weight (LMW) RAP1/RAP2 complex has been implicated in PVM formation (Ghosh et al., 2017); and the dense granule exported protein 1 (EXP1) and EXP2 form a nutrient-permeable channel in the PVM to take up nutrients such as amino acids and monosaccharides (Desai et al., 1993; Bullen et al., 2012; Garten et al., 2018; Iriko et al., 2018; Mesén-Ramírez et al., 2019). Once inside the erythrocyte, the parasite remodels the host cell to obtain nutrients required for development and division, and provides a means to evade the host immune system using erythrocyte surface variant antigens (Matthews et al., 2019). Protein export across the PVM is mediated by a channel assembled in the PVM, referred to as the *Plasmodium* translocon of exported proteins (PTEX) (de Koning-Ward et al., 2009). PTEX consists of EXP2, PTEX150, and heat shock protein 101 (HSP101) ATPase secreted from dense granules (Bullen et al., 2012; Beck et al., 2014; Elsworth et al., 2014). The rhoptry bulb high molecular weight (HMW) RhopH complex consisting of RhopH1/Clag, RhopH2, and RhopH3 is involved in the plasmodial surface anion channel (PSAC) formation for nutrient uptake through the erythrocyte membrane during the development of the intracellular parasite (Desai et al., 2000; Nguitragool et al., 2011; Counihan et al., 2017; Ito et al., 2017; Sherling et al., 2017; Gupta et al., 2018; Gupta et al., 2020).

RON3 in *P. falciparum* was identified as an orthologue of RON3 in *Toxoplasma gondii* (Bradley et al., 2005) and consists of 2215 amino acid residues with a signal peptide, three transmembrane domains, and a coiled-coil region (**Supplementary Figure 1A**) (Ito et al., 2011). RON3 is expressed in the schizont stage and is trafficked to the rhoptries in association with rhoptry-associated membrane antigen (RAMA) (Ito et al., 2011; Sherling et al., 2019). RON3 is localized at the body of the rhoptries, and not within the rhoptry neck as suggested by the name, and is discharged from the rhoptries into the PVM during invasion (Ito et al., 2011; Geoghegan et al., 2021). RON3 interacts with RON2, RON4, and RAMA (Ito et al., 2011; Ito et al., 2020). A global reverse genetics study indicates that the *P. falciparum* RON3 gene is essential for blood stage development (Zhang et al., 2018). Using conditional gene deletion by the DiCre recombinase system, the expression of partial RON3 N-terminal fragments causes a defect in protein export by PTEX and glucose uptake leading to the growth arrest of ring-stage parasites, but not the inhibition of prior invasion (**Supplementary Figure 1A**) (Low et al., 2019).

The full-length 260 kDa form of RON3 undergoes protein processing by plasmepsin IX (PMIX) to yield 190 kDa and 40 kDa truncated forms which are present in the rhoptries or nascent rhoptries (**Supplementary Figure 1A**) (Ito et al., 2011; Favuzza et al., 2020). It is not known which truncated form of RON3 is responsible for the above-described phenotypes, and at which developmental stage RON3 fragments act. To characterize the function of the RON3 C-terminal fragment and achieve stage-specific knockdown, we tagged RON3 with the *Escherichia coli* DHFR destabilization domain (DDD) at the C-terminal end. The DDD tag conditionally interferes with the target protein function as the fusion can be denatured by removing trimethoprim (TMP), a small stabilization molecule (Iwamoto et al., 2010). We found that disruption of the RON3 C-terminal fragment function is lethal for blood-stage parasites. TMP removal before RON3 expression causes a defect in erythrocyte invasion and growth arrest of ring-stage parasites. We show that the protein processing of full-length RON3 was partially abrogated in the schizont stage, and the RON3 C-terminal fragment could not transfer to ring-stage parasites. The export of dense granule protein by PTEX and glucose uptake through the PVM in ring-stage parasites just after invasion remained intact, but the export of secretory protein and glucose uptake in late ring-stage parasites were abrogated. Upon TMP removal after erythrocyte invasion, ring-stage parasites normally grew to the trophozoite stage. Our results suggest that the RON3 C-terminal fragment plays a role during erythrocyte invasion and secreting protein from the parasite plasma membrane to the PVM in ring-stage parasites.

## 2 Materials and methods

### 2.1 In vitro culture of *P. falciparum*

The *Plasmodium falciparum* 3D7 strain was cultivated at 37°C under 5% O_2_, 5% CO_2_, and 90% N_2_ in O+ human erythrocytes at 5% hematocrit in RPMI 1640 medium (Invitrogen) supplemented with 25 mM HEPES, 50 μg/mL hypoxanthine (Sigma-Aldrich), 2 g/L NaHCO_3_ (Gibco), 10 μg/mL gentamicin (Gibco), and 0.5% Microbiological Grade Bovine Albumin (MP Biomedicals).

### 2.2 Plasmid construction

**Figure 1:**
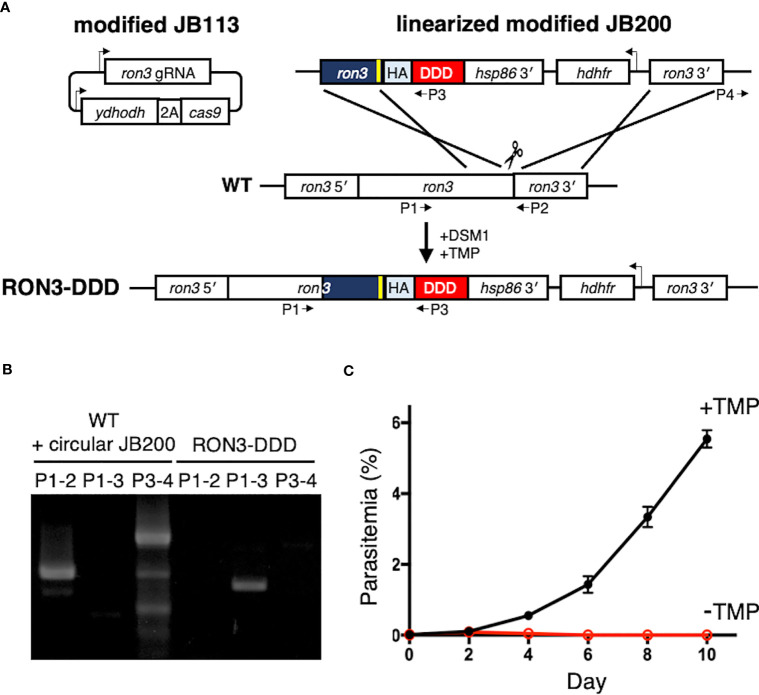
Conditional disruption of *P. falciparum* RON3 function. **(A)** Strategy for the introduction of a tandem 3xHA-DDD tag at the C-terminus of RON3 using the CRISPR-Cas9 system. The sgRNA of RON3 was expressed, and yeast dihydroorotate dehydrogenase (ydhodh) as well as *Streptococcus pyogenes* Cas9 (cas9) as a bicistronic transcript expressed and separated by a 2A viral skip peptide in the modified JB113 episome continuously maintained using the yDHODH drug-selectable marker. The linearized modified JB200 was inserted into the *ron3* gene locus by double crossover recombination. To repair the DNA double-strand break by Cas9, the modified JB200 carries two homology regions of the C-terminal of ron3 without a stop codon (navy blue) with the shield mutation (yellow) and 3′ UTR of ron3 (ron3 3′). HA, tandem 3x hemagglutinin; DDD, *Escherichia coli* DHFR destabilization domain; hsp86 3′, heat shock protein 86 terminator region. The primers (P1-4) were used to detect the wild type ron3 locus, integration of modified JB200, and circular modified JB200. **(B)** PCR for detection of the genomic sequence of wildtype RON3 (P1-2), integration of the donor sequence from the modified JB200 plasmid (P1-3), and the unexpected retention of circular modified JB200 plasmid (P3-4). **(C)** Replication of RON3-DDD parasites over the course of 4 to 5 erythrocytic cycles following asynchronous cultures with or without TMP. Parasitemia values were averaged from three biological replicate experiments and are presented as mean ± SEM.

Plasmids were designed to mediate CRISPR-Cas9 transfection of cultivated parasites to produce conditional knockdowns carrying a tandem 3xHA-DDD encoded as a C-terminal tag (**Figure 1A**). The JB113 plasmid (**Supplementary Figure 1B**) was digested at NotI sites flanking an HSP101 gRNA expression cassette. The modifiable sgRNA expression cassette was obtained by PCR using pL6-eGFP (Ghorbal et al., 2014) and inserted into the NotI-digested JB113 plasmid using Gibson Assembly cloning (New England BioLabs). The sgRNA of RON3 was inserted into the BtgZI-digested modified JB113 plasmid using In-Fusion cloning (Clontech). The yeast dihydroorotate dehydrogenase (yDHODH) and *Streptococcus pyogenes* Cas9 were expressed bicistronically by a 2A viral skip peptide, and sgRNA was expressed from the modified JB113 plasmid. DSM1 was used to retain the modified JB113 carrying yDHODH cassette (Ghorbal et al., 2014). The JB200 plasmid (**Supplementary Figure 1B**) was digested at XhoI and AvrII sites flanking genomic sequences of HSP101 for homologous recombination repair. Genomic sequences of RON3 for homologous recombination repair were obtained by PCR using genomic DNA as template and inserted into the digested JB200 plasmid carrying a tandem 3xHA-DDD tag using Gibson Assembly cloning. Shield mutation was introduced on the donor sequence at sites corresponding to the genomic sgRNA target using a codon-optimized primer. The modified JB200 plasmid carrying a human dihydrofolate reductase (hDHFR) cassette allows the selection of integrants with TMP. The primers used are listed in a **Supplementary Table 1**. DNA sequencing was used to confirm all constructed plasmids.

### 2.3 Transfection

Transfections were initiated by electroporation of 50 µg of modified JB113, and 20 µg of AflII digested linearized modified JB200 plasmids into 1.75 x 10^9^ uninfected erythrocytes prior to use for parasite cultivation. The Gene Pulsar II system (Bio-Rad) was used at 0.31 kV and 975 μF. Immediately after electroporation, 1 x 10^7^ schizonts purified by differential centrifugation on a 70%/40% Percoll-sorbitol gradient were mixed with electroporated erythrocytes and parasite culture was initiated. The transfected culture was selected with 1.5 µM DSM1 and 10 µM TMP to ensure retention of the JB113 plasmid and select the growth of transgenic parasites expressing hDHFR. After 2 to 3 weeks, parasite growth was detected by Giemsa staining of thin blood films. Parasite clones were obtained by limiting dilution (Lyko et al., 2012), and then PCR was used to evaluate the integration of the donor sequence from the modified JB200 plasmid and the unexpected retention of circular modified JB200 plasmid (**Supplementary Figure 1B**, **Supplementary Table 1**). All experiments were performed using parasite clones. Conditional knockdown was initiated by TMP removal before RON3 expression or after erythrocyte invasion.

### 2.4 Antibody production

A synthetic peptide was derived from the C-terminal repetitive sequence of ring-infected erythrocyte surface antigen in *P. falciparum* (PfRESA; PF3D7_0102200, EENVEENVEENVEENVEENV, with an additional N-terminal cysteine) and was conjugated with commercially procured keyhole limpet hemocyanin (KLH) carrier protein (Sigma-Aldrich). A Japanese white rabbit received a total of three immunizations with Freund’s complete and incomplete adjuvant at 3-week intervals (Kitayama Labes, Ina, Japan). Antisera was collected 14 days after the last immunization. The animal work was conducted by Kitayama Labes in compliance with the guidelines based on “Charter for Laboratory Animal Welfare” (Japanese Society for Laboratory Animal Resources). The anti-RESA antibody was verified for specific reaction to native 3D7 wild-type RESA by immunoblotting, indirect immunofluorescence assay, and immunoelectron microscopy (**Supplementary Figure 2**). The mouse anti-RESA monoclonal antibody 28/2 (Anders et al., 1987) was used as a positive control.

### 2.5 Immunoblots

Synchronous schizont-rich parasite cultures were used after membrane fractionation using hemolysis in lysis buffer (7.5 mM Na_2_HPO_4_, 1 mM EDTA, 1 mM PMSF, pH 7.5) and ultracentrifugation (100,000 x g, 4°C, 1 h). The supernatant was considered the soluble fraction, and the pellet was considered the membrane fraction. Ring-infected erythrocytes were lysed with 0.15% saponin in PBS containing cOmplete Protease Inhibitor Cocktail (Roche) for 10 min on ice. Samples were solubilized and reduced in a modified Laemmli sample buffer at a final 6% SDS concentration. Proteins were separated by electrophoresis in 5–20% polyacrylamide gels (ATTO) and transferred to PVDF membranes. Membranes were blocked using 3% skim milk in 150 mM NaCl, 20 mM Tris-HCl, pH 7.4 with 0.1% Tween 20. Each membrane was then immunostained at RT for 1 h with rabbit anti-HA antibody (Abcam), rabbit anti-RON3N antibody referred to as anti-PfRON3_1 (Ito et al., 2011), rabbit anti-RON2 antibody (Cao et al., 2009; Ito et al., 2011) for a schizont-specific control, rabbit anti-*Plasmodium* aldolase antibody (Abcam) as a protein loading control, and rabbit anti-RAP1 antibody (Ito et al., 2011) as a membrane protein loading control. Ponceau-S staining of PVDF membranes was used to visualize hemoglobin as a soluble protein loading control. Specific labeling was revealed by probing with horseradish peroxidase-conjugated secondary-antibody (ThermoFisher Scientific), and visualized with Immobilon Western Chemiluminescent HRP Substrate (Millipore) on an LAS 4000 Mini luminescent-image analyzer (GE Healthcare). The relative molecular masses of the proteins were estimated with reference to Precision Plus protein WesternC standards (Bio-Rad). Semi-quantitative analysis of the protein expression levels was performed by ImageJ and the loading controls were used for normalization.

### 2.6 Immunofluorescence microscopy

Indirect immunofluorescence assays were performed using air-dried thin blood smears of RON3-DDD parasites with or without TMP prepared side by side on microscope glass slides, fixed with ice-cold 100% acetone for 3 min, and stored at −80°C. The smears were thawed and blocked with PBS containing 3% skim milk at 37°C for 30 min. The antigen samples for IFA described above were stained with primary antibodies diluted at the following concentrations in blocking solution at 37°C for 1 h: rabbit anti-RESA antibody, 1:500; mouse anti-SBP1 antibody, 1:100 (Iriko et al., 2020); rabbit anti-EXP2 antibody, 1:500 (Miyazaki et al., 2021); mouse anti-EXP2 antibody, 1:100 (Morita et al., 2018). Secondary antibodies, Alexa Fluor 488-conjugated goat anti-rabbit IgG and Alexa Fluor 555-conjugated goat anti-mouse IgG (Invitrogen) or Alexa Fluor 488-conjugated goat anti-mouse IgG and Alexa Fluor 555-conjugated goat anti-rabbit IgG (Invitrogen), were used at a 1:500 dilution in blocking solution at 37°C for 30 min. DAPI (4’,6-diamidino-2-phenylindole) at 2 µg/ml was added to the secondary-antibody solution to stain the nuclei. Slides were mounted in ProLong Gold Antifade reagent (Invitrogen) and viewed under a 63× oil immersion lens. Images were collected on a confocal scanning laser microscope (LSM780; Carl Zeiss MicroImaging) with serial 405 nm, 488 nm, or 561 nm excitations with the same detector settings. Images were processed in Adobe Photoshop (Adobe Systems, CA, USA) or ImageJ. For differential counts of protein export pattern, a minimum of 100 infected cells were counted for each parasite stained with anti-RESA or anti-SBP1 antibodies.

### 2.7 Immunoelectron microscopy

Parasites were fixed for 30 min on ice in a mixture of 1% paraformaldehyde and 0.2% glutaraldehyde in HEPES-buffered saline (pH 7.05). Fixed specimens were washed, dehydrated, and embedded in LR White resin (Polysciences, Inc., Warrington, PA) as described (Aikawa and Atkinson, 1990; Ito et al., 2011). Ultrathin sections on the grids were blocked in PBS containing 5% non-fat skim milk and 0.01% Tween 20 (PBS-MT) at 37°C for 30 min. Grids were then incubated at 4°C overnight with rabbit anti-RESA or pre-immune sera in PBS-MT (1:500). After washing with PBS containing 4% BlockAce (Yukijirushi, Sapporo, Japan) and 0.01% Tween 20 (PBS-BT), the grids were incubated at 37°C for 1 h with goat anti-rabbit IgG conjugated to 15 nm gold particles (BBI Solutions) diluted 1:20 in PBS-MT, and rinsed with PBS-BT. The grids were then rinsed with distilled water, dried, and stained with uranyl acetate and lead citrate. Samples were examined with a transmission electron microscope (JEM-1230; JEOL, Tokyo, Japan).

### 2.8 Growth and invasion analysis

Comparison of growth of RON3-DDD parasites in the presence of TMP or DMSO was determined by microscopy of Giemsa stained thin blood films. For differential counts, a minimum of 2,000 erythrocytes were counted for each time point.

The invasive capacity of RON3-DDD parasites was determined by adding fresh erythrocytes to Percoll-purified schizonts to obtain a parasitemia of 0.3%. Parasitemia was determined by Hoechst 33342 staining (ThermoFisher Scientific) and analyzed by flow cytometry (LSRFortessa X-20; BD Biosciences) performed with the same detector settings. After 24 h, schizont rupture was monitored by microscopy of Giemsa stained thin blood smears and the cultures enabled the percentage of newly ring-infected RBCs to be similarly determined. Data were analyzed using FlowJo software (BD Biosciences).

Comparison of growth of RON3-DDD parasites after the merozoite invasion of erythrocytes in the presence of TMP or DMSO was determined by microscopy of Giemsa stained thin blood films. The viable free merozoites that retained their invasive capacity were purified based on published protocols (Boyle et al., 2010; Ito et al., 2013). Briefly, parasites were synchronized using sorbitol treatment. Late-stage parasites (32 to 36 h post-invasion) were purified using a Percoll-sorbitol gradient, incubated with 10 μM E64 (Sigma-Aldrich) for 6 h, and pelleted. E64-treated schizonts were resuspended in a small volume of incomplete culture medium and filtered through a 1.2-μm Acrodisc 32-mm syringe filter (Pall Corporation) onto fresh erythrocytes.

### 2.9 Live imaging of glucose analog uptake

Synchronized ring-stage parasites were stained with Glucose Uptake Probe-Green (Dojindo, Kumamoto, Japan) and 600 nM MitoTracker Deep Red (ThermoFisher Scientific) for 1 h at 37°C, after which the cells were washed with phenol red-free RPMI containing Washing and Imaging Solution (Dojindo). Cells were dispensed at 0.06% hematocrit to µ-Slide 8 well chamber slides (ibidi, Gräfelfing, Germany) coated with 0.01% poly-L-lysine (Sigma-Aldrich). Prior to imaging, Hoechst 33342 was added to the wells at a final concentration of 2 µM. Cells were imaged within 1 h by Zeiss LSM 780 with the same detector settings, and images were randomly collected by switching every five images between parasites in the presence of TMP or DMSO. The captured images were processed and analyzed using ImageJ. Mean fluorescence intensity (MFI) was measured by the fluorescence intensity attributed to the parasite (red circle as determined by localization with Hoechst/MitoTracker [blue/red] localization) and the erythrocyte (white circle), respectively, as shown in **Supplementary Figure 3**. Background MFI in the erythrocyte was subsequently subtracted from the MFI in the parasite to provide the change in MFI (ΔMFI).

### 2.10 Osmotic lysis measurements

Organic solute uptake by infected erythrocytes was continuously tracked as described (Nguitragool et al., 2011; Ito et al., 2017). Trophozoite-stage infected cells were Percoll-enriched after preincubation in 40% Percoll-sorbitol solution for 30 min at 37°C to allow efficient recovery of knockdown parasites that had reduced permeabilities (Ito et al., 2017). Enriched cells were washed, and resuspended in 150 mM NaCl, 20 mM Na-HEPES buffer, 0.1 mg/mL BSA, pH 7.4. Solute uptake was initiated by the addition of an osmotic lysis solution containing 280 mM sorbitol, 20 mM Na-HEPES, 0.1 mg/mL BSA, pH 7.4. The kinetics of osmotic lysis were monitored at 37°C for 1 h by recording the transmittance of 700 nm light through the cell suspension. The addition of 0.5% saponin at the end of each recording was used for the normalization of transmittance values to 100% cell lysis. Sorbitol permeability coefficients were calculated as 1/halftime of osmotic lysis.

### 2.11 Statistical analysis

All data were presented as mean ± standard errors of means (SEM). All quantitative data were statistically analyzed and graphed using GraphPad Prism 9 for Mac (GraphPad Software, MA, USA). Statistical significance was calculated by unpaired two-tailed Student’s t-test. Significance was accepted at p<0.05.

## 3 Results

### 3.1 The RON3 is essential for blood-stage parasites

To investigate the function of the RON3 C-terminal fragment, we tagged RON3 with a DDD at the C-terminal end using the CRISPR-Cas9 system (**Figure 1A**). Integrations were confirmed by PCR (**Figure 1B**). Using clones RON3-DDD carrying the C-terminal tag, we observed the effect of RON3 knockdown on parasite survival. When the DDD-stabilizing small molecule TMP was removed from asynchronous RON3-DDD cultures, a complete block in parasite growth was observed (**Figure 1C**).

### 3.2 DDD destabilization induces a defect in RBC invasion and the proteolytic processing inhibition of RON3 in schizonts

**Figure 2:**
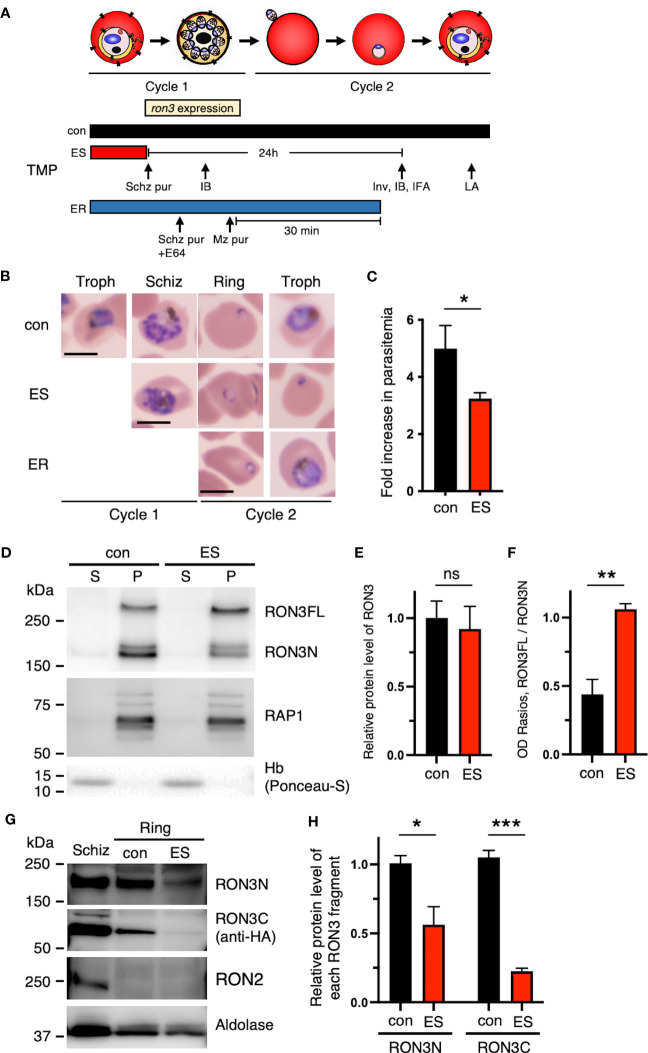
Destabilization of the DDD tag leads to protein processing inhibition of full-length RON3 in schizonts and the loss of the RON3 C-terminal fragment of ring-stage parasites. **(A)** Schematic showing the timeline for the stage-specific removal of TMP, *ron3* gene expression, and the subsequent harvest of infected RBCs for immunoblottings (IB), indirect immunofluorescence assays (IFA), invasion assays (Inv), and osmotic lysis assays (LA). Horizontal bars below the schematic indicate the presence of TMP. con, TMP is present continuously; ES, TMP is removed from early schizonts before *ron3* expression; ER, TMP is removed from early rings within 30 min after invasion. **(B)** Giemsa-stained micrographs of RON3-DDD at indicated intervals after TMP removal as shown in panel **(A)**. Bars, 5 µm. **(C)** Reduced ring formation by RON3 knockdown. The schizonts isolated from synchronous cultures with (con) or without TMP (ES) were incubated with fresh RBCs, and ring-stage parasitemias were determined by flow cytometry. Data were averaged from three biological replicate experiments. Statistical significance was determined by a two-tailed unpaired t-test where P < 0.05 is considered significant (*, P < 0.05). **(D)** Immunoblot of schizont-infected cell lysates from RON3-DDD parasites (con and ES) probed with anti-RON3N and anti-RAP1 antibodies (membrane protein loading control). Hemoglobin (soluble protein loading control) was probed with Ponceau-S. S and P, soluble and ultracentrifugation pellet fractions. **(E)** Semi-quantitative analysis of the protein expression levels of RON3 and **(F)** OD Ratios of the full-length RON3 to the RON3 N-terminal fragment in schizont stage parasites. Statistical significance was determined by a two-tailed unpaired t-test where P < 0.05 is considered significant (**, P < 0.01, non-significant (ns)). **(G)** Immunoblots of schizont or ring-infected cell lysates from RON3-DDD parasites (con and ES) probed with anti-RON3N, anti-HA, anti-RON2 (schizont-specific control), or anti-*Plasmodium* aldolase antibodies (loading control); and **(H)** semi-quantitative analysis of the protein levels of each RON3 fragment in ring stage parasites. Statistical significance was determined by a two-tailed unpaired t-test where P < 0.05 is considered significant (*, P < 0.05, ***, P < 0.001).

We next determined the stage-specific effects of RON3 knockdown on the asexual blood-stage developmental cycle (**Figure 2A**). Upon removal of TMP from the early schizont stage that expresses RON3 (ES, **Figure 2A**), RON3-DDD parasites completed their first intracellular development and invaded new erythrocytes accompanied by some reduction of parasitemia, and parasite development was arrested at the subsequent ring stage (ES, **Figure 2B**). To quantify the impact of RON3 knockdown on the invasion of erythrocytes, schizonts were isolated from synchronous cultures with or without TMP (con and ES, **Figure 2A**) and then added to fresh erythrocytes. The parasite nuclei were stained with Hoechst 33342, and a similar starting parasitemia was confirmed by flow cytometry (**Supplementary Figures 4A, C**). Following further incubation to allow schizont rupture and erythrocyte invasion, the parasitemia of newly-invaded ring-stage parasites was determined by flow cytometry as well as the starting parasitemia (**Supplementary Figures 4B, D**). Consistent with the growth retardation (**Figure 1C**), the parasites from cultures without TMP displayed significantly reduced ring formation, corresponding to 65% of control cultures with TMP (**Figure 2C**, **Supplementary Figure 3D**). We hypothesized that a reduction of the invasion rate upon RON3 knockdown might arise from the destabilized DDD interfering with the proteolytic processing of RON3-DDD. To investigate this, immunoblotting of the schizont lysates with an antibody against the RON3 N-terminal fragment was conducted (**Figure 2D**) and revealed that both the unprocessed 280 kDa protein (full-length RON3 (260 kDa) + 3HA-DDD tag (20 kDa)) and an 190 kDa cleavage product were similarly identified in the insoluble fraction of both TMP(+) and TMP(-) parasites (**Figure 2E**), but the ratio of full-length RON3 to the RON3 N-terminal fragment was significantly increased in TMP(-) parasites indicating 30% reduction of both N- and C-terminal fragments (ES, **Figure 2F**). Accordingly, conditional knockdown alters proteolytic processing but not protein expression level and solubility of RON3 in schizonts.

### 3.3 DDD destabilization induces the growth arrest of the ring-stage parasites and the loss of the RON3 C-terminal fragment in the ring-stage parasites but not the N-terminal fragment

TMP removal from the early schizont stage (ES, **Figure 2A**) induced not only a defect in erythrocyte invasion but also the growth arrest of the subsequent ring-stage parasites (ES, **Figure 2B**). To investigate the impact of DDD destabilization on the ring stage parasite growth, we next conducted immunoblotting of ring-stage parasite lysates with antibodies against the RON3 N-terminal fragment and 3xHA epitope tag (**Figure 2G**), and revealed an 80% reduction of the RON3 C-terminal fragment in TMP(-) parasites at the ring stage as compared to a 40% reduction of the RON3 N-terminal fragment (ES, **Figure 2H**), under conditions where RON2 was undetectable indicating no schizonts in the ring-stage parasite lysate (**Figure 2G**). In contrast, when TMP was removed in the early ring stage (0 to 30 min post-invasion) (ER, **Figure 2A**), RON3-DDD parasites normally grew to the trophozoite stage (ER, **Figure 2B**).

### 3.4 Knockdown of the RON3 C-terminal fragment does not alter dense granule protein export just after the merozoite invasion, but alters ring-stage expressed protein export

**Figure 3:**
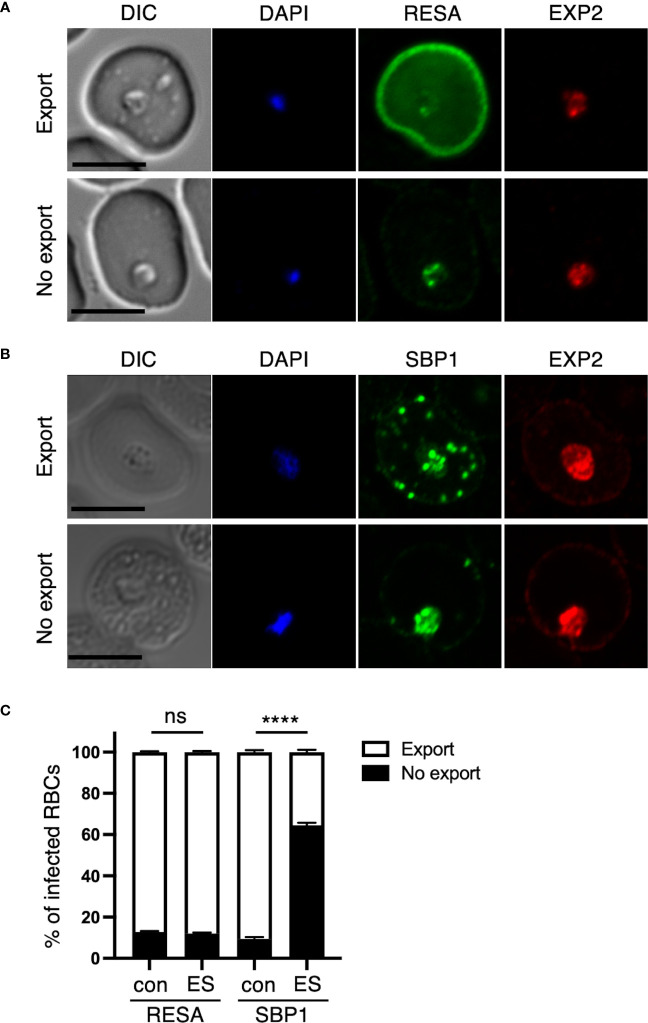
Disruption of the RON3 C-terminal fragment function leads to unusual protein export by PTEX translocon. Ring-infected RBCs from RON3-DDD parasites were cultivated with or without TMP (con and ES in **Figure 2A**). IFA images of ring-infected cells with **(A)** anti-RESA or **(B)** anti-SBP1 with anti-EXP2 antibodies. An image is shown for an export or no export pattern of A) RESA or B) SBP1. RESA and SBP1 staining are shown in green, and EXP2 staining is shown in red. The nuclei staining (DAPI) is shown in blue. DIC, differential interference contrast. Bars, 5 μm. **(C)** The percentages of each export pattern of RESA or SBP1. Data represent the mean ± SEM. Statistical significance was determined by a two-tailed unpaired t-test where P < 0.05 is considered significant (****, P < 0.0001, non-significant (ns)).

As TMP removal after erythrocyte invasion induces no phenotype within the intracellular development (ER, **Figure 2B**), we focused on the subsequent ring-stage parasites upon TMP removal from the early schizont stage (ES, **Figure 2B**). RON3-deficient parasites have a defect in protein export via PTEX (Low et al., 2019). To investigate the influence of the loss of the RON3 C-terminal fragment in ring-stage parasites on protein export, we examined distinct types of proteins exported by PTEX. Specifically, RESA is secreted from dense granules of merozoite as well as PTEX components at early ring stages (Riglar et al., 2011) and is exported by PTEX; whereas skeleton binding protein 1 (SBP1) is expressed in late ring stage parasites at 12 hours post-invasion (Blisnick et al., 2000) and is exported by PTEX as well as RESA. While RESA is normally distributed throughout the infected erythrocyte and is concentrated underneath the erythrocyte membrane (Export, **Figure 3A**), SBP1 is localized to Maurer’s clefts in the cytoplasm of ring-infected erythrocytes (Export, **Figure 3B**); however, the signal of each protein remains in close proximity to the parasite and is not translocated to the erythrocyte cytoplasm in 10% of infected cells (No export, **Figures 3A–C**) The percentages of export and no export pattern of each protein revealed a 50% reduction of SBP1 export in TMP(-) parasites (**Figure 3C**). Furthermore, the PTEX translocon component EXP2 was detected at the PVM in both parasites (**Figures 3A, B**).

### 3.5 Knockdown of the RON3 C-terminal fragment impairs glucose uptake in the ring stage

**Figure 4:**
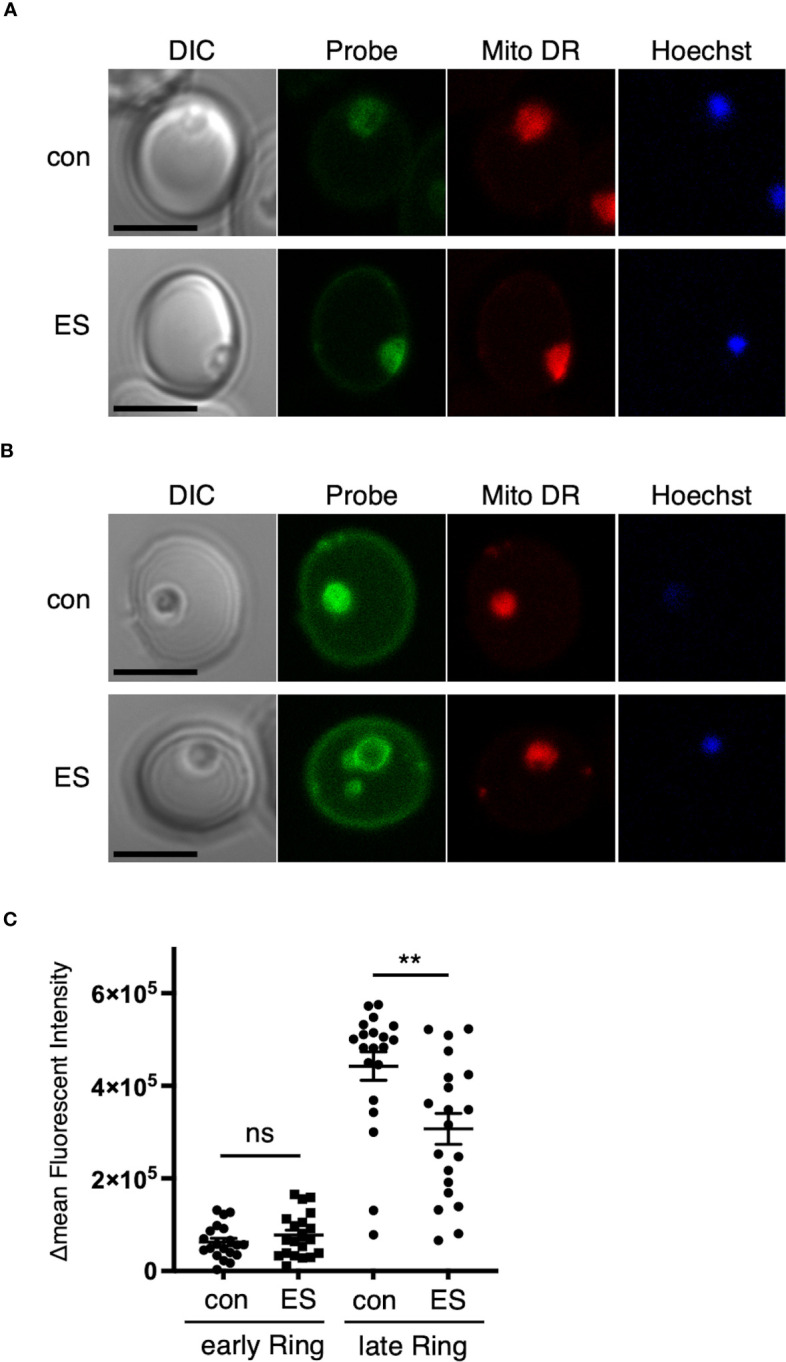
Disruption of the RON3 C-terminal fragment function leads to a defect in glucose uptake in the ring stage. Representative images of RON3-DDD parasites of **(A)** 0 to 6 or **(B)** 12 to 18 hours post-invasion cultivated with or without TMP (con and ES in **Figure 2A**) after 1 h of incubation with a labeled glucose analog. Parasites were additionally stained with Hoechst 33342 (DNA marker) and MitoTracker Deep Red (Mito DR), a mitochondrial marker. Bars, 5 μm. **(C)** ΔMFI of glucose analog in RON3-DDD ring-stage parasites of 0 to 6 or 12 to 18 hours post-invasion cultivated with or without TMP (n = 20/group). Data represent the mean ± SEM. Statistical significance was determined by a two-tailed unpaired t-test where P < 0.05 is considered significant (**, P < 0.01, non-significant (ns)).

Low et al. (2019) reported that RON3-deficient parasites exhibited a defect in glucose uptake, and so we investigated glucose uptake at different time points in ring-stage parasites. The synchronized second cycle of ring-stage parasite cultures with or without TMP were incubated with a labeled glucose analog. Cells were further stained with Hoechst 33342 and MitoTracker Deep Red, which allowed live parasites to be identified. In both experimental conditions at 0 to 6 hours post-invasion, there was no significant change in mean fluorescence intensity (ΔMFI) in glucose uptake (**Figures 4A, C**). At 12 to 18 hours post-invasion, a 31% significant reduction in glucose uptake into ring-stage TMP(-) parasites was observed (**Figures 4B, C**); indicating that the RON3 C-terminal fragment is important for glucose uptake through the PVM.

### 3.6 Knockdown of the RON3 C-terminal fragment affects PSAC activity in the trophozoite stage

**Figure 5:**
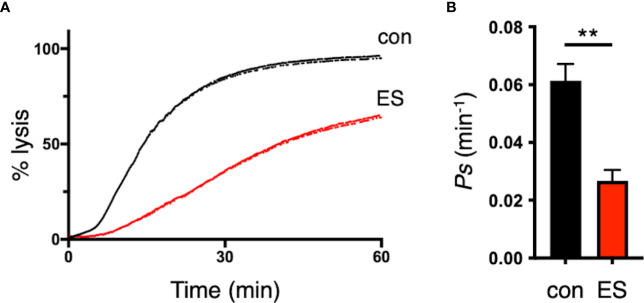
Disruption of the RON3 C-terminal fragment function leads to the reduction of PSAC activity. **(A)** Sorbitol-induced osmotic lysis kinetics of RON3-DDD parasites cultivated with or without TMP (con and ES in **Figure 2A**) (black and red traces, respectively). **(B)** Sorbitol permeability coefficients (Ps) were presented as mean ± SEM of the reciprocal of sorbitol-induced osmotic lysis halftime (t_0.5_) for control and RON3-DDD knockdown from three independent experiments. Statistical significance was determined by a two-tailed unpaired t-test where P < 0.05 is considered significant (**, P < 0.01).

Since the deletion of the RON3 C-terminal fragment in the ring stage leads to unusual parasite protein export through the PTEX translocon (**Figures 2G, H**, **3**), we next investigated if the reduction of trafficking of these parasite proteins in the infected erythrocyte leads to reduced nutrient uptake by the erythrocyte membrane PSAC. The kinetics of parasite nutrient uptake was measured by the osmotic lysis of infected erythrocytes in sorbitol, a sugar alcohol with primary uptake via PSAC (Pillai et al., 2010). Using synchronized parasites cultivated to the second cycle of the trophozoite stage with or without TMP, there was a significant 57% reduction in sorbitol uptake into the infected erythrocytes with RON3 knockdown parasites compared with control (p<0.01) (**Figures 5A, B**).

## 4 Discussion

In this study we have investigated the consequences of a RON3 post-translational knockdown on the parasite, to infer the function of the RON3 C-terminal fragment. We have shown that RON3 is essential for parasite survival and that disruption of the RON3 C-terminal fragment function from the early schizont stage results in a defect in erythrocyte invasion and growth arrest of subsequent ring-stage parasites. Knocking down the RON3 C-terminal fragment caused proteolytic processing inhibition of full-length RON3 in schizonts and the loss of the RON3 C-terminal fragment and retention of the RON3 N-terminal fragment in the ring stage parasites, and unusual abrogation of protein export by the PTEX translocon and glucose uptake at the PVM. We focused on investigating the function of the RON3 C-terminal fragment via a knockdown approach that is distinct from the work reported by Low et al. (2019). Therefore, we will mainly focus on discussion with regard to the results of RON3-deficient parasites which express partial N-terminal fragments (**Supplementary Figure 1A**).

First, in this study erythrocyte invasion is significantly affected by the knockdown of the RON3 C-terminal fragment from the early schizont stage (ES, **Figure 2C**). RON3-deficient parasites express small partial N-terminal fragments without the C-terminal region (Low et al., 2019) (**Supplementary Figure 1A**), while RON3-DDD parasites express both N- and C-terminal fragments in schizonts. Therefore, it is possible that other protein(s) complement the RON3 C-terminal fragment function in the RON3-deficient parasites. We also showed that destabilization of the DDD tag caused the proteolytic processing inhibition of full-length RON3 and leads to the reduction of both RON3 fragments in schizonts. Since the DDD tag is fused with the C-terminus of RON3, this suggests that the RON3 C-terminal fragment could contribute to erythrocyte invasion.

Secondly, we showed that RON3-DDD parasites export RESA normally (**Figure 3C**), suggesting that the PTEX translocon is active. In contrast, Low et al. (2019) showed that their RON3-deficient parasites, expressing only partial N-terminal fragments, had a deficiency of RESA export. Thus, it is suggested that the RON3 N-terminal fragment is essential for the formation of the active PTEX translocon at the PVM or the escort of exported protein from the PV to PTEX. However, it is unclear why most of the SBP1 signal remained in the RON3-DDD parasites (**Figure 3C**). RESA and PTEX components are secreted from the dense granules into the PV and reach the PVM just after the invasion (Riglar et al., 2011); but SBP1 is expressed in late ring-stage parasites at 12 hours post-invasion (Blisnick et al., 2000). The RON3 C-terminal fragment could participate in the ring-stage expressed protein export or play a role in the trafficking of those proteins from the parasite plasma membrane to the PVM.

Thirdly, in both studies, nutrient uptake is affected by RON3 knockdown. Glucose uptake at the PVM in RON3-deficient parasites at the ring stage was decreased but not completely lost (Low et al., 2019): The RON3-DDD parasites showed the reduction of glucose uptake in the late ring stage but not in the early ring stage (**Figure 4C**), indicating that EXP2 and EXP1 are secreted from the dense granules into the PV after invasion and form a nutrient-permeable channel in the PVM in an independent role of the PTEX complex (Bullen et al., 2012; Garten et al., 2018; Iriko et al., 2018; Mesén-Ramírez et al., 2019). Why glucose uptake was abrogated as ring-stage parasites develop is unknown; EXP2 and EXP1 are newly expressed in late ring-stage parasites (Bullen et al., 2012; Garten et al., 2018; Chappell et al., 2020) and increase the nutrient-permeable channel in the trophozoite-stage parasites (Mesén-Ramírez et al., 2019). As SBP1 was not exported in the RON3-DDD parasites, the RON3 C-terminal fragment might be involved in the trafficking of EXPs expressed in late ring-stage parasites from the parasite plasma membrane to the PVM. On the other hand, the nutrient uptake through the erythrocyte membrane by PSAC in RON3-DDD parasites was decreased but not wholly lost (**Figure 5**), suggesting that the PSAC-related RhopH complex is also secreted from the rhoptries into the PV and exported into erythrocytes by the PTEX translocon. However, completion of PSAC activity might need additional export of the RhopH complex or other exported proteins.

The RON3-DDD parasites arrested in development from the ring to the trophozoite stage (ES, **Figure 2B**), suggesting that RON3 could be important for nutrient uptake. Reducing nutrient-permeable channel activity in the PVM rather than PSAC activity may contribute to the growth arrest of ring-stage parasites. Genetic interference of EXP2 or EXP1 leads to the growth arrest of ring-stage parasites and diminishment of nutrient-permeable channel activity in the PVM (Garten et al., 2018; Mesén-Ramírez et al., 2019). On the other hand, it has been shown that loss of RhopH2 or RhopH3 function leads to growth arrest of schizont-stage parasites and affects PSAC but not nutrient-permeable channel activity in the PVM (Counihan et al., 2017; Ito et al., 2017; Sherling et al., 2017).

We used the DDD tag to distinguish the roles of the RON3 C-terminal fragment at distinct developmental stages; and we could not observe a defect in the single-cycle intracellular development of RON3-DDD parasites upon TMP removal just after invasion (ER, **Figure 2B**). Kinetic studies reveal that this approach permits post-translational knockdown in *P. falciparum*, and it is limited by a halftime of 6 hours for protein destabilization (Muralidharan et al., 2011). We previously showed that the DDD knockdown of RhopH2 immediately after the RBC invasion induced 40% reduction of PSAC activity in the trophozoite stage (Ito et al., 2017). The timing of the RON3 knockdown likely is sufficient to investigate the function of RON3 after invasion. Thus, the function of the RON3 C-terminal fragment before or within 6 hours after the invasion might be necessary for ring-stage parasite progression.

In conclusion, disrupting the RON3 C-terminal fragment function is lethal for blood-stage parasites. The role of the RON3 C-terminal fragment in erythrocyte invasion that we report in this study provides additional motivation for drug or vaccine development. Although we need further analysis of the RON3 C-terminal fragment and its interacting molecules in both the schizont and the subsequent ring stage, specific inhibitors interacting with one or more members of those RON3 complexes may disrupt disease progression by inhibiting both parasite invasion and nutrient acquisition, and as a partner drug kill the dormant ring-stages of artemisinin-resistant parasites.

## Data availability statement

The original contributions presented in the study are included in the article/**Supplementary Material**. Further inquiries can be directed to the corresponding authors.

## Ethics statement

The studies involving human participants were reviewed and approved by the Ethical Review Committee of Faculty of Medicine, Tottori University (Approval number: 21A201). Written informed consent for participation was not required for this study in accordance with the national legislation and the institutional requirements. The animal study was reviewed and approved by Kitayama Labes Co., Ltd. (Ina, Japan). All animal immunizations were commercially conducted at Kitayama Labes Co., Ltd. according to their Ethical Guidelines for Animal Experiments.

## Author contributions

DI and HO conceived and designed experiments. All authors conducted experiments. DI and HO analyzed the data. DI and HO wrote the manuscript. All authors contributed to the article and approved the submitted version.
