# Supplementary material for: Roles of the RON3 C-terminal fragment in erythrocyte invasion and blood-stage parasite proliferation in Plasmodium falciparum
Source: Front Cell Infect Microbiol. 2023 Jun 29;13:1197126. doi: 10.3389/fcimb.2023.1197126 (PMC10340547; doi:10.3389/fcimb.2023.1197126)
Supplement: Supplementary file 1 [file DataSheet_1.pdf]

frontiers

Supplementary Material

Roles of the RON3 C-terminal fragment in erythrocyte invasion and blood-stage parasite proliferation in *Plasmodium falciparum*

Daisuke Ito*, Yoko Kondo, Eizo Takashima, Hideyuki Iriko, Amporn Thongkukiatkul, Motomi Torii, Hitoshi Otsuki*

*** Correspondence:** Daisuke Ito: dito@tottori-u.ac.jp, Hitoshi Otsuki: otsuki@tottori-u.ac.jp

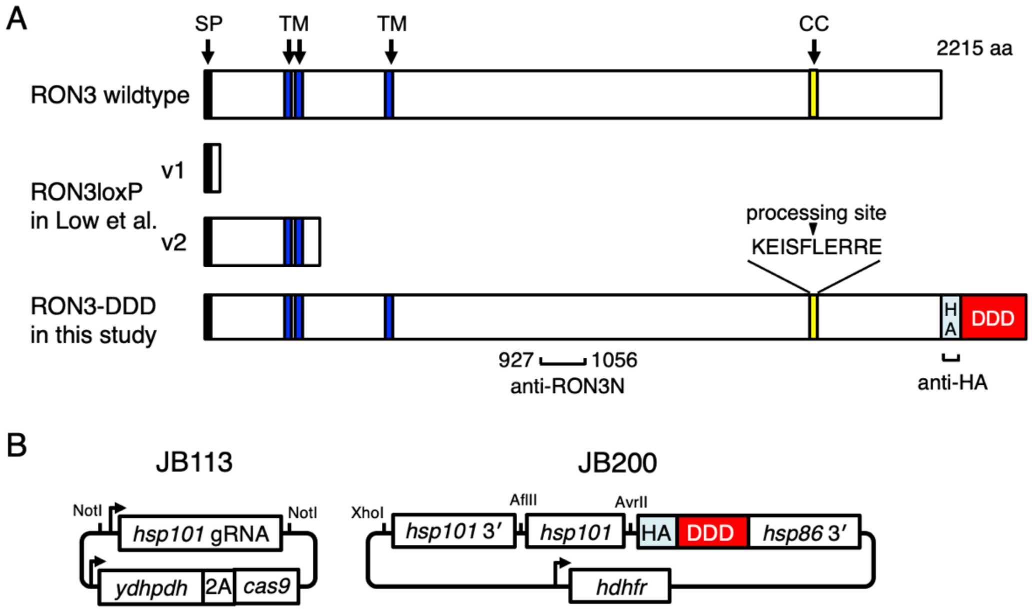

Supplementary Figure 1. A) Schematic representation of the primary structure of each RON3 in 3D7 wildtype, RON3loxP v1 and v2 in Low et al., and RON3-DDD in this study. SP and TM indicate putative signal peptide (black) and transmembrane (blue) sequences, respectively. The yellow box indicates a coiled-coil region, including the processing site by plasmepsin IX. The regions reacted with anti-RON3N, and anti-HA antibodies are indicated. B) JB113 and JB200 plasmid. JB113 plasmid carrying sgRNA of HSP101 and yDHODH-2A-Cas9 expression cassette. JB200 plasmid carrying genomic sequences of HSP101 for homologous recombination repair, a tandem 3xHA-DDD tag, and an hDHFR expression cassette.

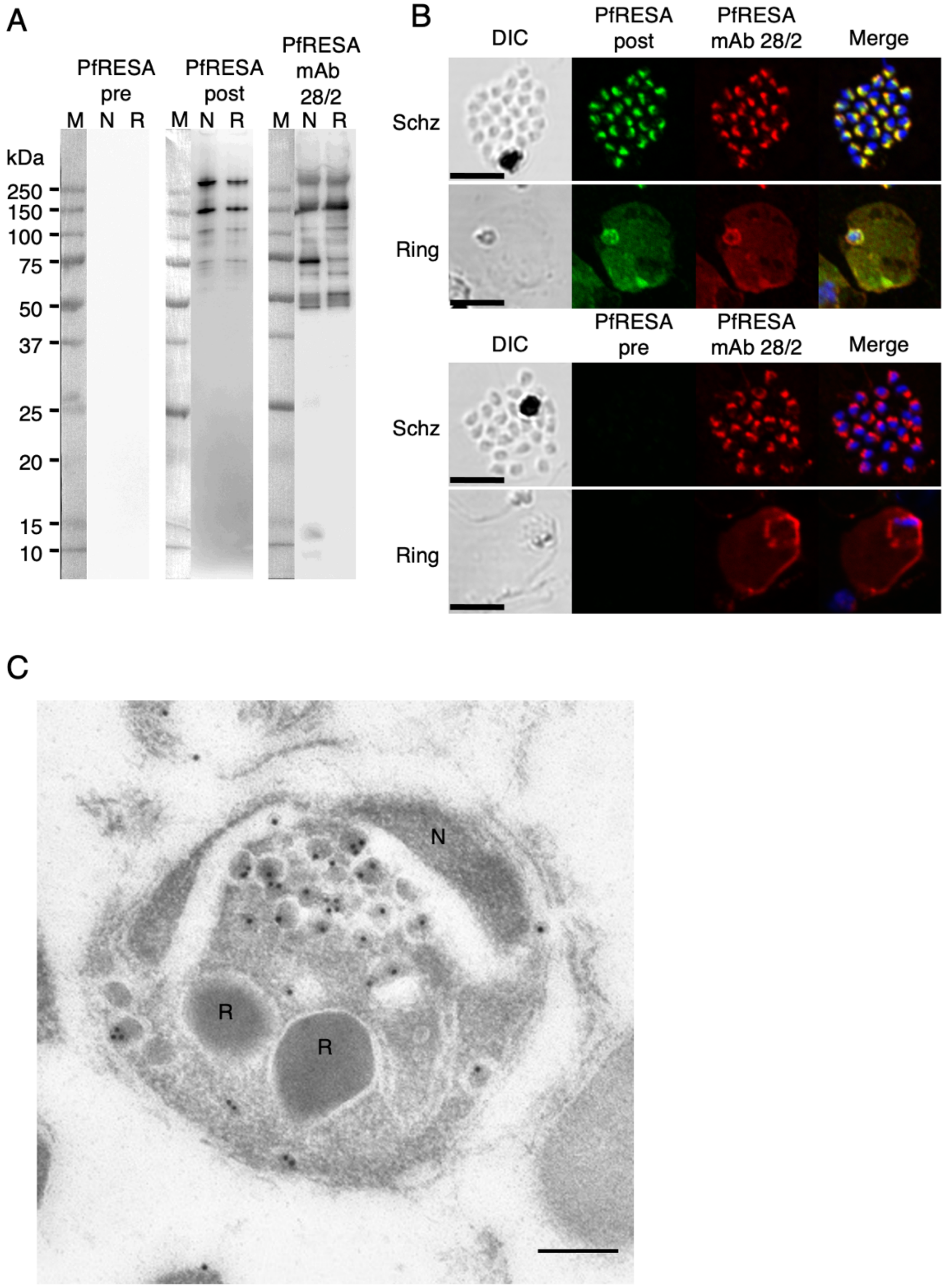

Supplementary Figure 2. Anti-RESA rabbit antibody production. A) Immunoblots of schizont-infected cell lysates from 3D7 wildtype probed with rabbit anti-RESA pre- or post-immune serum or mouse monoclonal antibodies against PfRESA. N and R, non-reducing and reducing condition. M, marker. B) IFA images of schizont or ring-infected cells with rabbit anti-RESA pre- or post-immune serum or mouse monoclonal antibodies against PfRESA. DIC, differential interference contrast. Merge, combination with green, red, and blue (DAPI). Bars, 5 µm. C) Dense granule localization of PfRESA by immunoelectron microscopy. Longitudinally sectioned merozoites in mature schizonts were labeled with rabbit anti-PfRESA antibodies followed by secondary antibodies conjugated with gold particles. The image shows that the gold particle signals were restricted to the merozoite dense granule. N and R, nuclei and rhoptry. Bar, 200 nm.

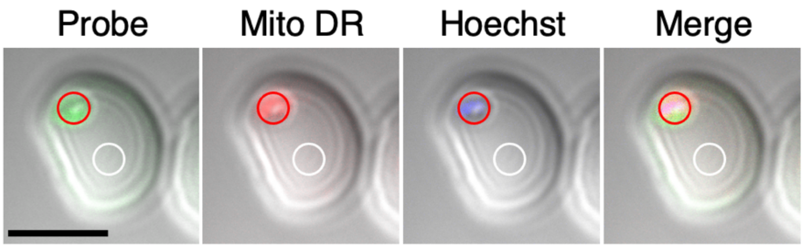

Supplementary Figure 3. Example illustration of a strategy for determining the change in mean fluorescent intensity (ΔMFI) of Glucose Uptake Probe-Green. MFI (green) was measured by first measuring fluorescent intensity attributed to the parasite (red circle as determined by localization with Hoechst 33342/MitoTracker Deep Red [blue/red] localization). Background MFI in the blood cell where the parasite resides (white circle) was subsequently subtracted to provide ΔMFI. Bars, 5 µm.

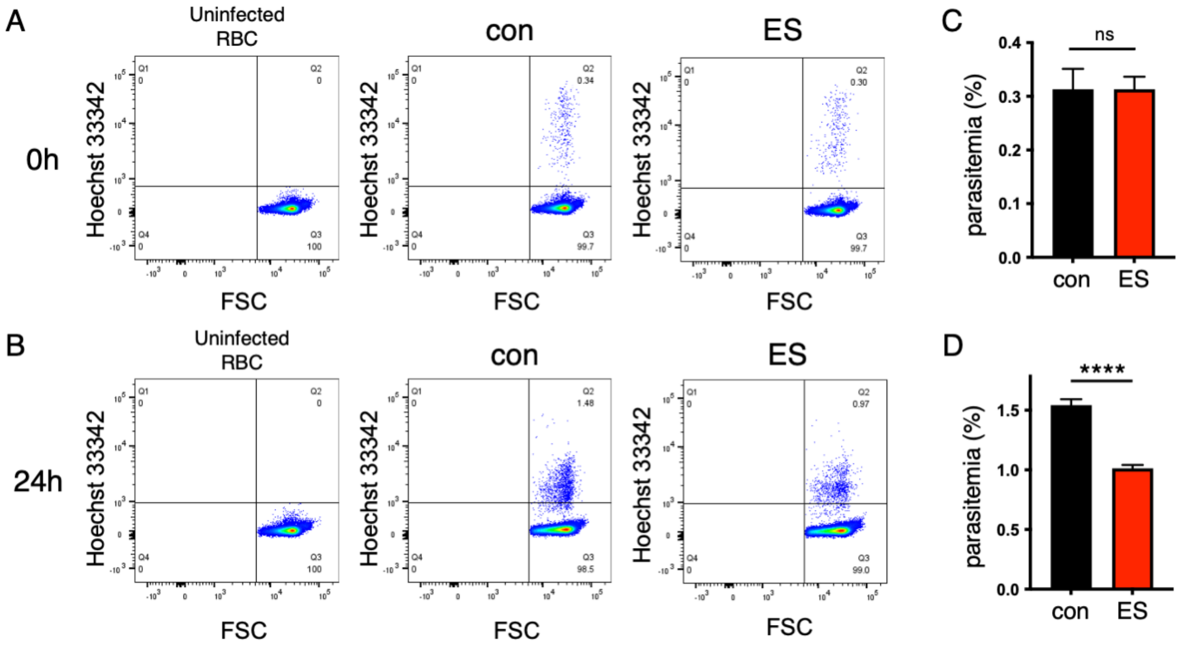

Supplementary Figure 4. Representative flow cytometry gating strategy to define parasitemia of A) schizonts at starting point and B) ring-stage parasites after 24 h in RON3-DDD parasites cultivated with or without TMP (con and ES in Figure 2A). The y axis represents the Hoechst 33342 signal, and the x axis represents the forward scatter (FSC). The *P. falciparum*-infected RBCs are shown in the gate Q2 on the top right. Parasitemia values of C) schizonts and D) ring-stage parasites were averaged from three biological replicate experiments and are presented the mean ± SEM. Statistical significance was determined by a two-tailed unpaired t-test where $P < 0.05$ is considered significant (****, $P < 0.0001$, non-significant (ns)).
